# Detection, Purification and Elucidation of Chemical Structure and Antiproliferative Activity of Taxol Produced by *Penicillium chrysogenum*

**DOI:** 10.3390/molecules25204822

**Published:** 2020-10-20

**Authors:** Ashraf El-Sayed, Gamal Enan, Abdul-Raouf Al-Mohammadi, Ahmed H. Moustafa, Nashwa El-Gazzar

**Affiliations:** 1 Botany and Microbiology Department, Faculty of Science, Zagazig University, Zagazig 44519, Egypt; gamalenan@ymail.com (G.E.); mora_sola1212@yahoo.com (N.E.-G.); 2 Chemistry Department, Faculty of Science, Zagazig University, Zagazig 44519, Egypt; almohammadi26@hotmail.com; 3 Department of Sciences, King Khalid Military Academy, Riyadh 11495, Saudi Arabia; ah_hu_mostafa@hotmail.com

**Keywords:** *Penicillium chrysogenum*, taxol, instrumental analysis, antiproliferative activity

## Abstract

*Penicillium chrysogenum* has been reported as a potent taxol producer based on quantitative analysis by TLC and HPLC. The biosynthetic potency of taxol has been validated from PCR detection of rate-limiting genes of taxol synthesis such as taxadienesynthase and 10-de-acetylbaccatin III-O-acetyltransferase (DBAT), which catalyzes the immediate diterpenoid precursor of the taxol substance, as detected by PCR. Taxol production by *P. chrysogenum* was assessed by growing the fungus on different media. Potato dextrose broth (PDB) was shown to be the best medium for obtaining the higher amount of taxol (170 µg/L). A stepwise optimization of culture conditions necessary for production of higher amounts of taxol was investigated. The substance taxol was produced optimally after 18 d of incubation at 30 °C in PDB adjusted initially at pH 8.0 with shaking (120 rpm) (250 µg/L). The *P. chrysogenum* taxol was purified successfully by HPLC. Instrumental analyzes such as Fourier transform infrared spectroscopy (FTIR), ultraviolet (UV) spectroscopy, ^1^HNMR and ^13^C NMR approved the structural formula of taxol (C_47_H_51_NO_14_), as constructed by ChemDraw. The *P. chrysogenum* taxol showed promising anticancer activity.

## 1. Introduction

Natural agents have recently shown a promising potential to be used as therapy for either treatment of cancer or inhibition of multidrug pathogenic bacteria [1,2,3,4,5,6,7,8,9]. They also possess an attractive property in that they are safe agents. In this regard, taxol (paclitaxel) is an oxygenated diterpenoid; it was firstly separated from the bark of Pacific yew tree (*Taxus brevifolia*) and showed anticancer activity [10,11]. It was reported previously that taxol showed a promising activity against ovarian cancer, breast cancer, non-small cell lung cancer, AIDS-related Kaposi’s sarcoma, head and neck carcinoma and other cancer types [11,12]. Taxol blocks cell proliferation by elevating the constancy of microtubules at the G2-M phase of the cell cycle and in turn prevents their dissolution to tubulin [10,12,13]. Restricted availability of mature yew trees, delayed growth of cultivated plants and depressed yield of the taxol substance has resulted in rising its cost and has raised attention to find other anticancer therapies. This makes taxol an investment load for many patients [14]. Therefore, there is a need to continue research to find out other natural alternative ways to produce taxol from other sources [10,13,15]. Research work focused recently on discovering taxol-producing strains of endophytic fungi that are able to produce taxol as an extracellular metabolite. It was approved that these fungal isolates contain the genes encoding 10-de-acetylbaccatin III-O-acetyltransferase (DBAT), which is the precursor necessary for taxol biosynthesis [16,17].

Thus, many researchers reported production and characterization of taxol from fungi, which are a promising prospect as a cheap method of production via industrial fermentations [18,19,20,21]. It was reported recently that many endophytic fungal species can produce taxol [22]. Due to mandatory use of taxol as an anticancer agent, the potential to produce it from cheap ways using fungal fermentation and to concur with the recent search to find out other sources of the expensive taxol, this work was undertaken to search for other taxol-producing fungi. Production and characterization of taxol from one strain of *P. chrysogenum* was reported; anticancer activity of the produced taxol was evaluated.

## 2. Results

**Figure 1:**
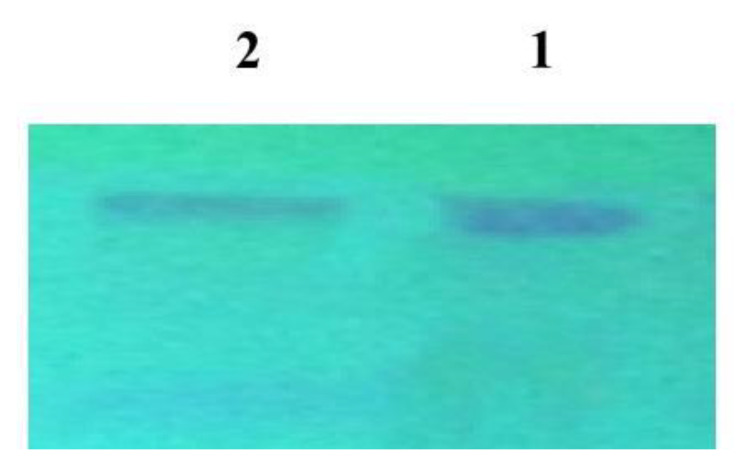
Chromatographic analysis of the extracted taxol using TLC analysis: Lanes 1 and 2. A band of about 500 bp was shown, indicating detection of 10-de-acetylbaccatin III-O-acetyltransferase (DBAT) genes which encode for taxol production by *P. chrysogenum.*

**Figure 2:**
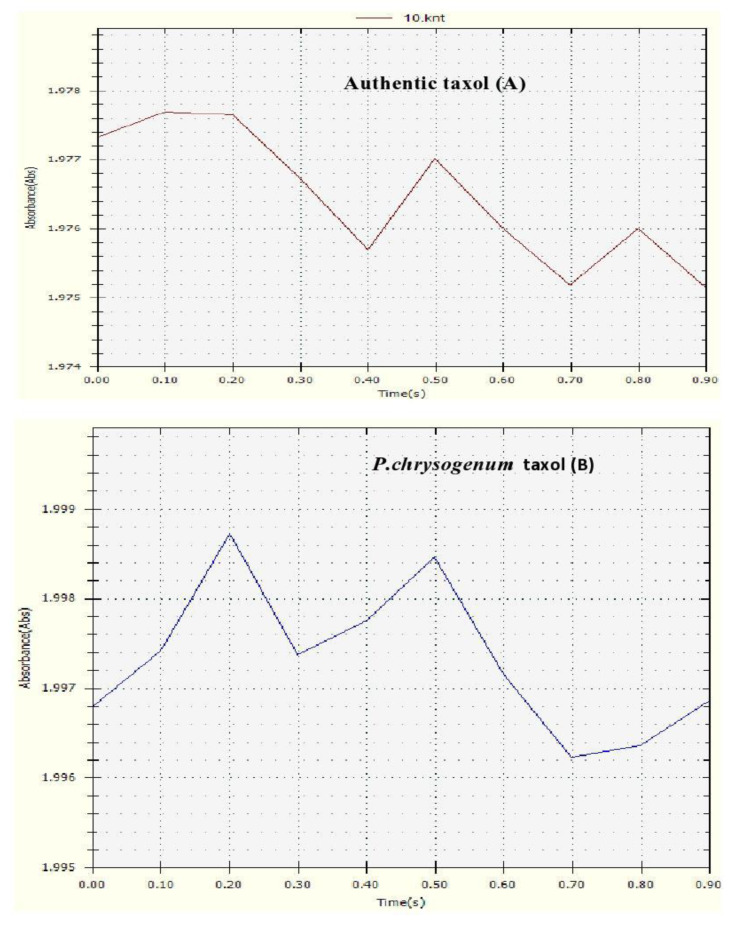
UV absorbance profiles at 227 nm of both authentic taxol (**A**) and *P.chrysogenum* taxol (**B**).

Out of thirty fungal isolates tested, only one (no.16) gave the highest taxol yield that has been identified as *P. chrysogenum*. Growth of *P. chrysogenum* and taxol production was compared in different media: PDB was showed to be the best medium for obtaining the maximum yield of taxol (170 µg/L) (*p* value < 0.05) (Supplementary Table S1). CFS was collected after growth of *p. chrysogenum* (10^5^ spores/mL) in PDB medium as given in Materials and Methods. The extracts were fractionated by TLC and showed similar chromatographic properties to the standard taxol. Partially purified taxol obtained from TLC showed a single dark blue spot with an R_f_ value similar to that of the standard taxol (Figure 1). *P. chrysogenum* gave the potentiality to produce the suspected taxol substance at 227 nm by absorption analysis with an optical density at 1.99 ± 1.80 which was similar to that of the standard taxol at 1.97 ± 1.78 (Figure 2).

**Figure 3:**
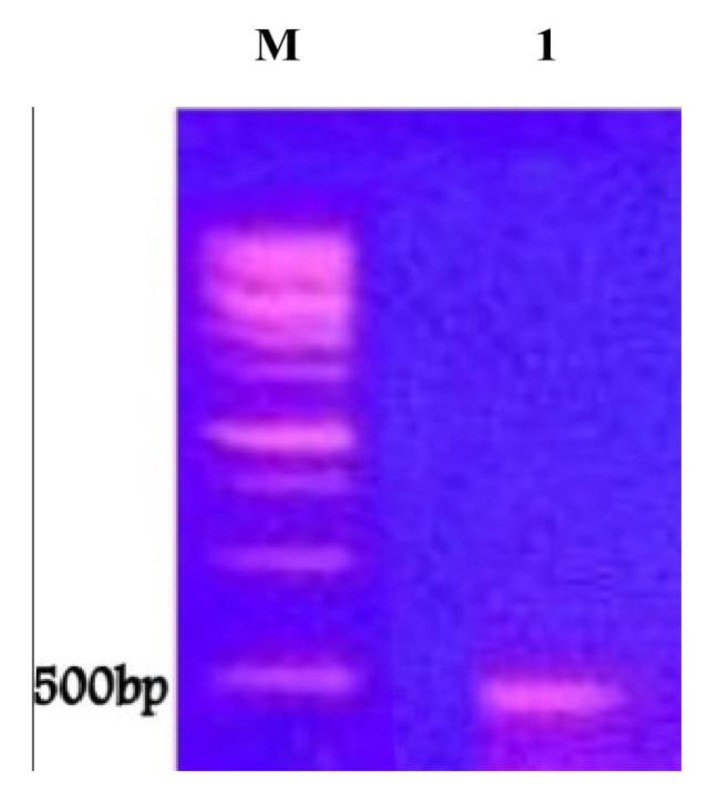
Agarose gel electrophoresis of the PCR product of the 10-deacetylbaccatin III-O-acetyltransferase (DBAT), the precursor necessary for taxol biosynthesis. Lanes M and 1 refer to marker DNA and *P. chrysogenum* taxol gene, respectively.

To confirm at the molecular level whether the identified strain *P. chrysogenum* contains the genes encoding the precursor of taxol production (DBAT), a PCR test was carried out as described in Materials and Methods. The PCR product was taken and electrophoresed via agarose gel (Figure 3). A band of about 500 bp was shown, indicating detection of DBAT genes which encode taxol production by *P. chrysogenum*.

**Figure 4:**
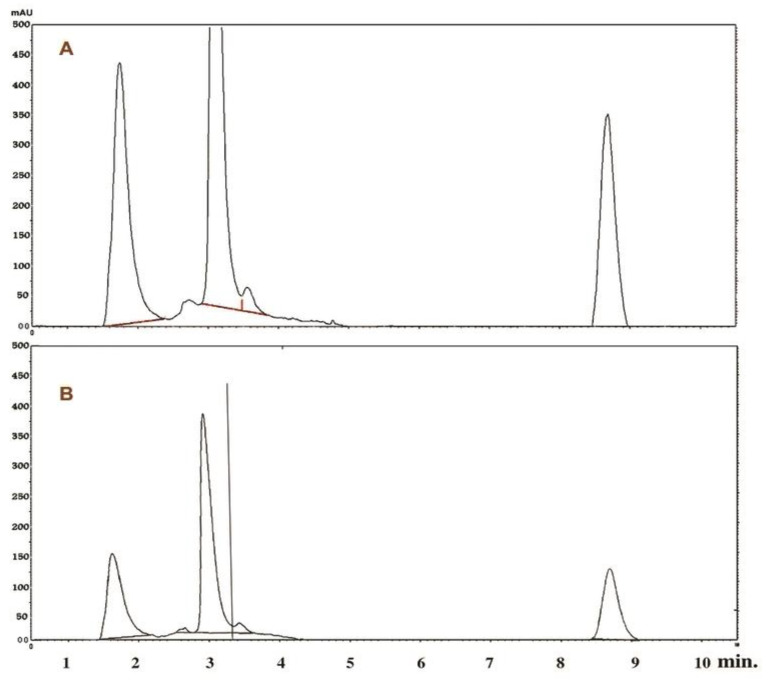
HPLC profiles of authentic taxol (**A**) and *P. chrysogenum* taxol (**B**). A peak with a retention time of 8.2 min was detected in authentic taxol and *P. chrysogenum* taxol.

To study optimization of growth conditions necessary for production of taxol by *P. chrysogenum*, PDB was used as a basal medium. Biosynthesis of taxol by *P. chrysogenum* was studied with function of time. Results are given in Table 1. The biosynthesis of taxol started almost after one week of incubation of culture at 30 °C; taxol production increased by a further incubation period, reaching its maximum values (187–200 µg/L) in the time range (16–18 d); the maximum yield of taxol (200 µg/mL) was detected after 18 d of incubation (*p* value < 0.05). Taxol production by *P. chrysogenum* decreased by further incubation >18 d. The effect of the initial pH value on *P. chrysogenum* growth and taxol production was studied. The highest values of growth and taxol production were achieved by *P. chrysogenum* in PD broth adjusted initially at an initial pH value 7–8 (200–220 µg/L) (*p* value < 0.05) and incubated at 30 °C (230 µg/L) by agitation speed at 120 rpm (Table 1) (250 µg/L) (*p* value < 0.05). After injection of 1 µg/mL of authentic taxol and *p. chrysogenum* taxol into HPLC equipment at the C18 column, a peak area with a retention time of about 8.2 min and 8.2 min, respectively, were shown, indicating the purity of *P. chrysogenum* taxol as compared to authentic taxol (Figure 4A,B).

**Figure 5:**
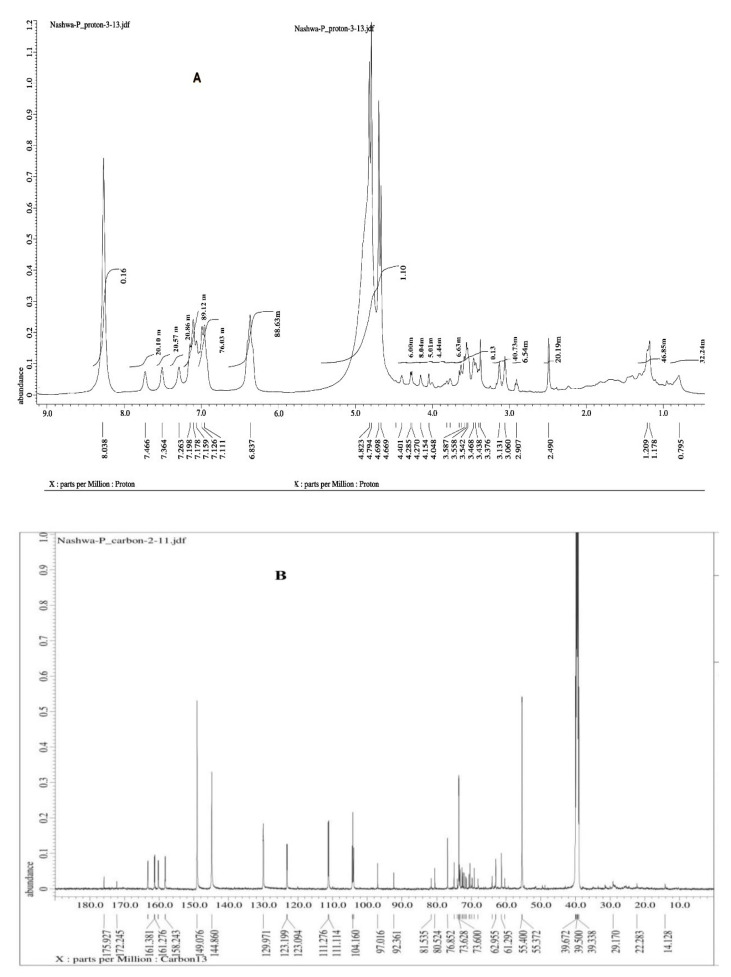
NMR spectra of the *P. chrysogenum* taxol. (**A**) refers to ^1^H NMR; (**B**) refers to ^13^C NMR.

The chemical structure of the purified taxol from *P. chrysogenum* was confirmed by ^1^H NMR, ^13^C NMR and FTIR analyzes. The resolved signals of ^1^HNMR for *P. chrysogenum* taxol were identical as compared to authentic taxol; the signals were distributed between 1.0 and 8.0 ppm (Figure 5A). Three proton signals were resolved at 1.0–2.5 ppm corresponding to methyl, acetate and acetylene groups, while signals for aromatic moieties were resolved at 7.0–8.4 ppm. H^1^ NMR (DMSO.d_6_ ): δ = 0.79 (s, 3H, CH_3_, CH_17_), 1.17 (s, 3H, CH_3_, C_16_), 1.20 (s, 3H, CH_3_, C_19_), 2.20 (s, 1H, C_6_, β^−^), 2.49 (s, 3H, CH_3_, C_18_), 3.06 (s, 3H, OCOCH_3_, C_10_), 3.13 (S, 3H, OCOCH_3_, C_4_), 3.37 (S, 1H, C_14_ (β^−^)), 3.39 (s, 1H, C_14_(α^−^)), 3.43 (s, 1H, OH, C_7_), 3.46 (s, 1H, C6(α)), 3.54 (s, 1H, OH, C_2_^−^), 3.55 (s, 1H, C_3_), 3.58 (s, 1H, C_20_(β^−^)), 4.04 (s, 1H, C_20_ (α^−^)), 4.66 (s, 1H, C_2_), 4.69 (s, 1H, C_7_), 4.79 (s, 1H, C_5_), 4.82 (s, 1H, C_2_), 5.11 (s, 1H, C_3_^−^), 5.13 (s, 1H, C_13_), 5.22 (s, 1H, C_10_), 6.83 (s, 11H, N_3_^−^), C_2_^−^COPh, 7.11 (s, 2H, O^−^), 7.12 (s, 2H, M^−^), 7.17 (s, 1H, P^−^) and 8.03 (s, 5H, Ar-H, C_3_).

^13^C NMR spectra of the taxol sample acquired in solution showed a chemical shift assignment (Figure 5B). In the results obtained (Figure 5B), the spectrum ^13^C NMR (DMSO-d_6_) δ = 14.12, 22.28, 29.17, 39.338, 39.500, 39.672, 55.372, 55.40, 60.32, 61.29, 62.95, 73.25, 73.44, 73.60, 73.62, 74.03, 74.99, 76.85, 80.52, 81.53,81.55 (sp^3^-carbon atoms), 103.9, 104.1, 104.3, 111.1, 111.2, 123.0, 123.1, 129.9, 144.8, 149.0, 158.2, 158.3, 160.2, 161.2, 161.28 163.2, 172.24 and 175.9 (sp^2^-carbon and 4 C=O, amid, ketone and esters). ^13^C NMR revealed the presence of 47 carbon atoms signals assigned with the molecular formula of taxol C_47_.

**Figure 6:**
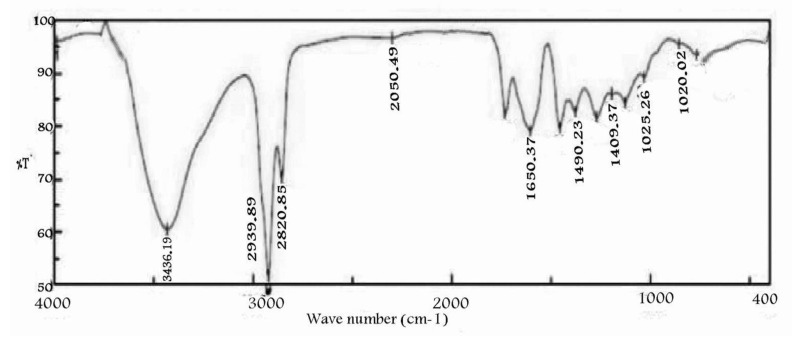
FTIR analysis for the extracted *P. chrysogenum* taxol.

The taxol obtained showed the same FTIR peaks of authentic taxol (Figure 6). The peak at almost 3436 cm^−1^ was very broad hydroxyl (3OH) and amide NH. By contrast, the peaks at almost 2939, 2820 and 2050 cm^−1^ were assigned to the aliphatic CH stretch, ester group stretch and aromatic rings stretch, respectively. The peak at almost 1650 cm^−1^ was assigned to keto groups(C=O, amide). The peaks at almost 1490 and1409 cm^−1^ were assigned for aromatic CH_3_ bending. The COO- stretching frequency peaked at almost 1268 cm^−1^. The -O- ester peaked at almost 1150 cm^−1^, while the peak at almost 1025 cm^−1^ was assigned for the aromatic CH bends.

**Figure 7:**
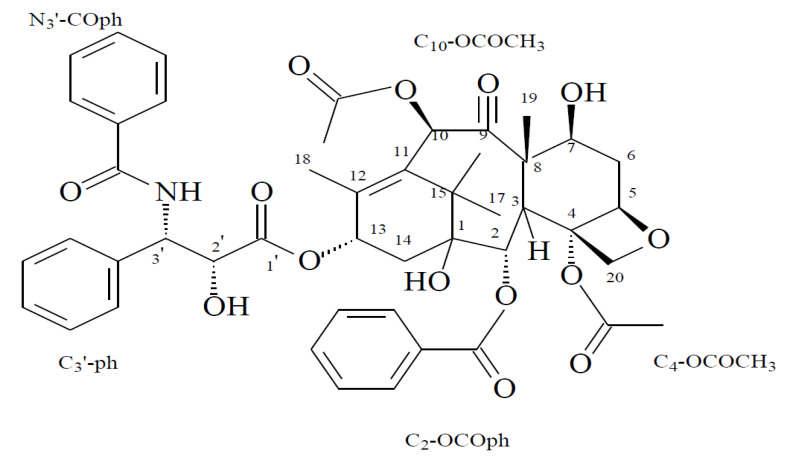
The structural formula (C_47_H_51_NO_14_) of the purified *P.chrysogenum* taxol with molecular weight at 853.9 as illustrated by ChemDraw.

The structure of the obtained taxol by *P. chrysogenum* was elucidated by ChemDraw using the results obtained. It was proposed as C_47_H_51_N O_14_, Mwt.: 853.9 (Figure 7).

**Figure 8:**
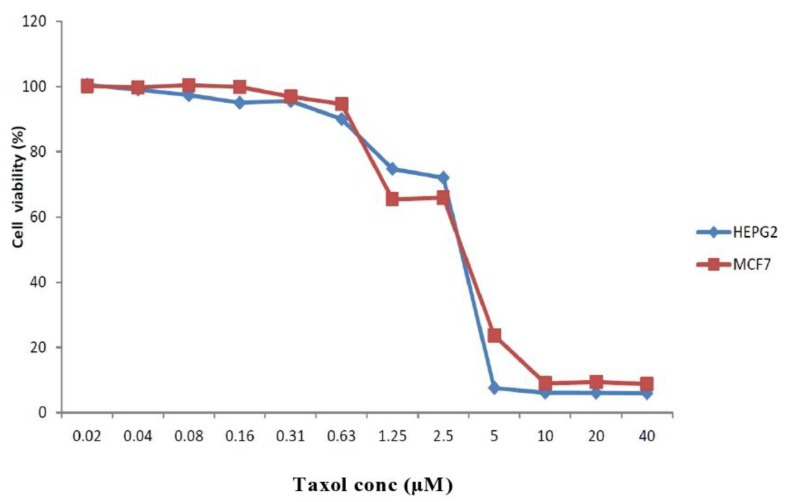
Antiproliferative activity of the *P.chrysogenum* taxol against liver tumor cells (HEPG 2) and breast adenocarcinoma (MCF 7) viability.

**Figure 9:**
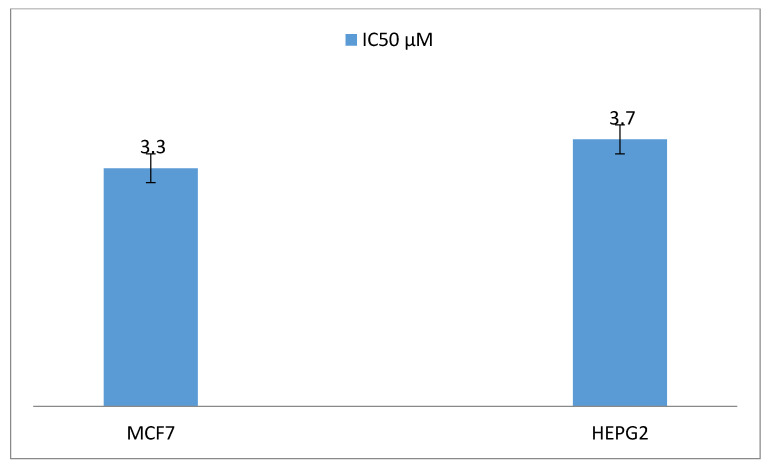
IC_50_ values of the *P. chrysogenum* taxol against liver tumor cells (HEPG2) and breast adenocarcinoma (MCF7).

Antiproliferative activity of the taxol obtained was evaluated against various cell lines such as liver tumor cells (HEPG2) and breast adenocarcinoma (MCF7) viability (Figure 8). A significant effect of *P.chrysogenum* taxol was shown (*p* value < 0.05) as cell viability of both MCF7 and HEPG2, which decreased distinctively by increasing *P.chrysogenum* taxol concentrations (Figure 8). The purified *P.chrysogenum* taxol has IC_50_ values of about 3.3 and 3.7 µM towards MCF7 and HEPG2, respectively (Figure 9).

## 3. Discussion

Taxol showed a promising activity against many types of cancers [14]. Fungal taxol-producing potency has raised the hope for obtaining crucial treatment of all types of cancers as they are produced by fungal fermentation by large amounts and with cheap costs [15,23]. This showed that there is a need to continue research to find out other fungal strains to produce taxol with promising amounts. In this regard, this study aimed to search for new fungal strains that are capable of producing taxol with high amounts. Out of 30 fungal strains isolated from rhizosphere region of leguminous plants, only one of them (no. 16) that identified as *P. chrysogenum* was shown to produce taxol. Such an isolate was obtained from the rhizosphere region of the plant soy bean (*Glycine max).*

Production of taxol by rhizospheric fungi is possible as both plant roots and rhizospheric soils might be colonized by fungi, giving plant–fungi interactions in the rhizosphere which can improve agricultural ecosystems [24].

Taxol production was reported previously by endophytic fungi [24,25,26]. The isolation of endophytic fungal strains needs a plant host; their growth can face some difficulties and need high costs [24,27]. Therefore, isolation and characterization of taxol-producing fungi from soil is needed. In this regard the obtained strain herein (R16 strain) was isolated from the rhizosphere region of the plant *Glycine max* and this will be a promising and interesting prospect for taxol production with cheap costs.

Taxol was detected in CFS preparations of *P. chrysogenum* as an absorbance peak, which was obvious at 227nm and which coincided with that of authentic taxol. This is in agreement with published work in this respect [10,13,25].

Taxol biosynthesis, as a secondary metabolite, by fungi is encoded by a cluster of genes (DBAT genes). The 10-de-acetylbaccatin III-10-O-acetyl transferase (DBAT) is more of a diagnostic marker for taxol biosynthesis [23,27]. Thus, to further validate this result, DBAT genes were amplified by PCR and used as the molecular marker for confirmation of the genetic linkage of taxol. In the present study, PCR screening showed positive results for DBAT genes with the genome of *P. chyrsogenum*-producing taxol; such results confirmed that *P. chrysogenum* possessed the DBAT genes and this corresponds with published work in this respect [23,28].

The results obtained showed that the PDB medium gave the best value of the suspected taxol by *P. chyrsogenum.* Previous results showed that the PD medium plays an important role in enhancement of taxol productivity [29,30]. The crude sample from the silica gel showed a partially purified compound that has chromatographic properties similar to that of the standard taxol on TLC [31]. From the HPLC chromatogram, the chemical identity of putative taxol sample of *P. chrysogenum* was in agreement with the corresponding sample from *Bartalinia robillardoides* [31,32].

In the presented results, stepwise optimization of taxol production by *P. chrysogenum* was studied aiming at optimization of growth conditions of the producer strain. The optimum incubation time was detected in the range of 16–18 d after of incubation. This is coupled with previously published work in this regard [30,32] and is dissimilar to results obtained by other authors [33]. The highest production of taxol after 18 d of incubation approved that the taxol produced is a secondary metabolite and fully produced at the end of lag phase of fungal growth [34]. The *P. chrysogenum* taxol was optimally produced after 18 d of incubation of broths adjusted initially the pH range 7–8.0. This corresponds with previously published work [30,35]. At this initial pH value (8.0), the best incubation temperature was 30 °C and this is similar to that reported previously [30]. The agitation rate necessary for *P. chrysogenum* taxol was checked at the above optimum rate for further growth conditions, which was 120 rpm, as reported previously [34]. On the other hand, 150 rpm was used for maximum taxol production by *A. fumigatus* [35]. The agitation rate affects venting quality where a low agitation rate caused a small venting effect, which is not favorable for fungal growth. However, too high an agitation rate may induce autolysis of mycelium [36,37].

Interestingly, the results of TLC and HPLC analyzes approved taxol production by *P. chrysogenum* and confirmed the success of the results obtained by PCR amplification of DBAT gene as rate-limiting genes of taxol biosynthesis; the purity of *P. chrysogenum* taxol was additionally approved by HPLC [17,31,32]. The chemical structure of the extracted taxol from *P. chrysogenum* was confirmed by ^1^H NMR, ^13^C NMR and FTIR analysis. The resolved signals of ^1^HNMR for *P. chrysogenum* taxol samples were identical to those of the authentic one and the signals were distributed between 1.0 and 8.0 ppm. Three proton signals were resolved at 1.0–2.5 ppm corresponding to methyl, acetate and acetylene groups [18,36], while the signals for aromatic moieties were resolved at 7.0–8.4 ppm. Consistently, for all Taxane scaffolds, signals for their side chains protons were resolved at 2.0–7.0 ppm, while those for benzoate (C2), phenyl (C3) and benzamide (C3) groups were resolved at 7.0 and 8.4 ppm [26,38]. Moreover, the chemical identity of *P. chrysogenum* taxol was further confirmed from the ^13^CNMR signals that were identical to authentic taxol [26]. Taxol of *P. chrysogenum* has the same FTIR peaks obtained for authentic taxol. The peaks at 3436 cm^−1^ were assigned for the hydroxyl (OH) and amide (± C (O) NH ±) group stretches [10,13,39]. While, the peaks at 2993 and 2820 cm^−1^ were assigned to the aliphatic CH stretch, ester group stretch and aromatic rings stretch, respectively [40,41]. The COO stretching frequency was peaked at 1490 cm^−1^, while the peak at 1025 cm^−1^ was assigned for the aromatic CH bends [10,40].

The anticancer activity of *P. chrysogenum* taxol was evaluated against HEPG2 and MCF7 with a strong cytotoxic activity for MCF7 and HEPG2 cell lines (IC_50_ 3.3–3.7 µM). *P. chrysogenum* taxol has a more cytotoxic activity against HEPG2 and MCF7 tumor cells than *Cladosporium oxysporum* (430 µM) extracted taxol [26,40]. In addition, the cytotoxic activity of *P. chrysogenum* taxol against HEPG2 and MCF7 tumor cells is strongly higher than the taxol obtained from *T. brevifolia* (690–892 µM) [42], (714–690 µM) [12] and (702–892 µM) [42].

## 4. Materials and Methods

### 4.1. Isolation and Characterization of Fungi Suspected to Produce the Taxol Molecule

Thirty fungal isolates were isolated from the rhizosphere region of many leguminous plants cultivated in Sharkia Governorate (100 km north Cairo), Egypt; they were assayed for taxol production as reported previously [43]. The isolate number 16, which was isolated from the rhizosphere region of the plant soybean (*Glycin max*), was shown to produce the suspected taxol substance. This isolate grew on different media, viz., malt extract agar, yeast sucrose agar, Czapek–Dox agar and potato dextrose agar (all from Sigma) and its cultural characteristics were recorded as reported previously [18,44,45]. Spore characteristics and morphology of hyphae were studied by microscopic examination of fungal hyphae stained with cotton blue in lactophenol. This isolate was characterized as belonging to *Penicillium chrysogenum* and designated as *P. chrysogenum* R16 (*P. chrysogenum)*. Slope cultures of this R16 strain were prepared onto potato dextrose agar and stored in a refrigerator at 4 °C throughout the experiments. For inocula preparation, slope growth in test tubes was flooded with sterile peptone water with 0.1% Tween 20 and was gently scratched with a sterile needle loop; the inoculum was adjusted by aid of a hemocytometer at about 10^5^ spores per milliliter [43,46].

### 4.2. Determination of the Taxol Substance in Cell Free Supernatant (CFS) of P. chrysogenum

*P. chrysogenum* was inoculated into PD broth (10^5^ spores/mL); cultures were incubated at 30 °C for 15 d as this is the incubation period necessary for production of secondary metabolites by fungi [20]. The cultures were then filtered throughout four layers of cheesecloth to remove mycelia necessary for DNA isolation to be used in further experiments. To the culture filtrate, 0.025% *w*/*v* Na_2_CO_3_ was added with frequent shaking in order to reduce the amount of fatty acids that may contaminate the substance taxol in the culture. The Na_2_CO_3_ treated culture filtrate was filter-sterilized using Millipore filters (0.25 µm, Millipore, Filters, Amicon, Mumbai, India), designated as culture free supernatants (CFS) and used for further experiments. This CFS was extracted with double volume of dichloromethane (DCM). The organic extract was then evaporated using a rotary evaporator (IKA, RV10, Staufen im Breisgau, Germany) at 35 °C; the dried residue was resuspended in 3 mL methanol and analyzed using the necessary chromatographic and spectroscopic methods [21,47].

To detect the molecule taxol, TLC analysis (qualitative separation) was used [21,48]. TLC precoated silica gel plates (20 × 20 cm) were prepared (TLC Silica gel 60 F_254,_ Merck KGaA, Darmstadt, Germany). These TLC plates were developed with the solvent system (methylene chloride/methanol/dimethyl formamide) (90:9:1, *v*/*v*/*v*). After running, they were examined using UV illumination at 227 nm (Min-UVIS, DESAGA, Heidelberg, Germany). In addition, the plates were sprayed with 1% vanillin in sulfuric acid (*w*/*v*) with gentle heating [48,49]. A bluish spot fading to dark grey was developed after 24 h, compared to authentic taxol (Sigma-Aldrich, Cat. # T7402, St. Louis, MO, USA). Putative spots of silica containing taxol were scraped off from the plate at the appropriate R_f_ value and dissolved in methylene chloride and vortexed thoroughly for 3 min. The presence of taxol was identified by comparison with standard taxol. The absorption spectrum of taxol separated from the CFS of *P. chrysogenum* on the TLC plate was recordedinaUV4 Unicam UV/Vis spectrometer (ATIUnicam, Cambridge, UK) and compared with that of the authentic taxol [50].

### 4.3. Detection of Genes Encoding Taxol Production within P. chrysognum Genome

Total DNA was extracted from *P. chrysogenum* after its growth in potato dextrose broth (PDB) for 15 d [44,46,47]. The primers used for amplification of genes encoding 10-de-acetylbaccatin III-O-acetyltransferase (DBAT) that catalyze the immediate diterpenoid precursor of taxolwere5′-GGAGCCAATATGGAAGAGAGAAT-3′ as forward primer and 5′-TCCATG TTGCACGAGACTTAC-3′ as reverse primer (Promega Company, Madison, WI, USA) [43]. PCR amplification was carried out in a Gene-AMP PCR system 9600 thermocycler (Promega Company, Madison, WI, USA). The PCR reaction mixture contained 1 μL of either primer and completed to 20 μL with sterile distilled water. Amplification conditions were 94 °C for 10 min and 35 cycles for denaturation. Then 95 cycles for 30 s annealing extension were performed at 56 °C for 1 min. The PCR product was cleaned up using a gene JET™, PCR purification Kit, Germany, (Fermntase), electrophoresed via agarose gel (1.2%) (Promega Company, Madison, WI, USA) and visualized throughout using a gel documentation system (New Brunswek Scientific Company, NJ, USA) [44,51].

### 4.4. Optimization of Growth Conditions Necessary for Taxol Production by P. chrysogenum

In this set of experiments, PDB appeared to be the best medium for obtaining the highest values of growth and taxol production by *P.* *chrysogenum* and was used as a basal medium. Aliquots of PDB (250 mL for each) in 500 mL Erlenmeyer flasks (Gomhuria Company, Zagazig, Egypt) were inoculated by *P.* *chrysogenum* spores (10^5^ spores/mL) at their final concentration. Culture conditions necessary for taxol production were stepwisely optimized with regard to different incubation times (20–30 d), different initial pH values (2.0–9.0), different incubation temperatures (20–40 °C) and at different agitation rates (90–200 rpm). CFS and fungal biomass were prepared as described above, and taxol was determined as described herein [18,48].

### 4.5. High Performance Liquid Chromatography (HPLC) of P. chrysogenum Taxol

The purity and concentration of the extracted taxol were analyzed by HPLC (Agilent Technology, G1315D, Santa Clara, CA, USA) using a C18 reverse phase column (Eclipse Plus C18 4.6 × 150 mm, 3.5 μm, Cat.# 959963-902) with isocratic mobile phase of methanol/acetonitrile/water (25:35:40, *v*/*v*/*v*) at a flow rate of 1.0 mL/min for 20 min. The sample and mobile phase were filtered through a 0.2 µm filter (Millipore, Bilters, Amicon, Mumbai, India) before injecting into the column. Taxol fractions were scanned from 200 to 500 nm by a photodiode array detector. Their chemical identity and concentrations were confirmed from the retention time and absorption peak area at 227 nm. The taxol was confirmed by comparing the peak area of the fungal samples with that of standard taxol [52,53].

### 4.6. Instrumental Analysis of the Suspected Taxol Substance

The purified taxol substance by HPLC from the above steps was subjected to instrumental analysis [48,49,50,51,52]. Fourier transform infrared (FTIR) and ultraviolet (UV) spectra of the suspected taxol molecule were carried out as described previously in the microanalytical center, Cairo University (Egypt) [13,53]. ^1^H and ^13^C NMR spectra were carried out (JEOL, ECA-500II, 500MHz NMR, Faculty of Science, Mansoura University, Mansoura, Egypt) in comparison to authentic taxol. Samples were dissolved in Deut-rated chloroform (CDCl_3_), chemical shifts were given in ppm (δ-scale) and coupling constants were expressed in hertz (Hz) [52,54].

### 4.7. Antiproliferative Activity of the Taxol Substance Produced by P. chrysogenum

Both liver carcinoma (HPG2) and breast carcinoma (MCF7) were prepared (VACERA Institute, Cairo, Egypt). The viability of these tumor cells was assessed by 3-(4,5-dimethylthiazol-2-yl)-2, 5-diphenyl tetrazolium bromide (MTT) assay [55,56]. A 96-well plate was prepared, seeded with 10^3^ cells/ well and then incubated for 24 h at 35 °C. Cells were then supplemented with taxol (0.02–40 µM) and re-incubated for 48 h at the same incubation temperature. MTT dye (20 μL) was added to each cell, incubated for 6 h, and the purple color of the developed formazan complex was measured at λ570 nm [55,56]. The IC_50_ value was expressed by the taxol concentration that reduced the growth of 50% of an initial number of tumor cells.

### 4.8. Statistical Analyzes

All experiments were conducted in triplicates and results were expressed using one-way ANOVA analysis for estimating means and standard deviations (±SD). The test was followed by the least significant difference (LSD) test with statistical WASP software version 2.0; LSD, at significant level (*p <* 0.05).Sample symbols (a.a): mean non-significant difference; (a.b): mean significant difference [57].

## 5. Conclusions

The substance taxol was shown to be produced by *Penicillium chrysogenum* R16. This taxol substance was produced in potato dextrose modified at optimum growth conditions and was purified using TLC and HPLC chromatographic analyzes. Instrumental analyzes such as UV, NMR and FTIR identified the chemical formula (C_47_H_51_NO_14_) of the taxol produced; this taxolsubstanceshowed promising anticancer activity.

## Figures and Tables

**Table 1:** Effect of growth conditions on taxol production by *P.chrysogenum.*

	Value Applied	Taxol µg/L
	4	0.0
	6	50 ^h^ ± 4.0
	8	85 ^g^ ± 5.0
	10	110 ^f^ ± 10.0
	12	150 ^d^ ± 5.0
	14	170 ^c^ ± 5.0
	16	187 ^bc^ ± 5.0
	18	200 ^a^ ± 20.0
	20	195 ^b^ ± 5.0
	22	187 ^bc^ ±0.0
	24	160 ^d^ ± 2.0
	26	130 ^e^ ± 10.0
	28	80 ^gh^ ± 10.0
	30	40 ^i^ ± 5.0
**pH Value**	2	0.00
	3	20 ^f^ ± 2.0
	4	65 ^e^ ± 3.5
	5	100 ^d^ ± 1.0
	6	150 ^c^ ± 5.0
	7	200 ^b^ ± 2.0
	8	220 ^a^ ± 5.0
	9	95 ^d^ ± 5.0
**Incubation** **Temperature (°C)**	20	100.31 ^c^ ± 0.0
	25	150.43 ^c^ ± 10.0
	30	230.92 ^a^ ± 0.0
	35	200.1 ^b^ ± 10.0
	40	60.86 ^d^ ± 0.0
**Agitation Speeds (rpm)**	Static	150.47 ^c^ ± 0.0
	90	200.27 ^bc^ ± 10.0
	120	250.21 ^a^ ± 5.0
	150	210.51 ^a^ ± 0.0
	200	200.93 ^ab^ ± 10.0

The mean calculated was carried out for triplicate measurements from two independent experiments. ± SD means with different letters in the same column are considered statistically different (LSD test, *p* value < 0.05).

## Supplementary Materials

The following are available online: Table S1. Effect of different media on growth and taxol production by *P.chrysogenum.*
