# Supplementary material for: Detection, Purification and Elucidation of Chemical Structure and Antiproliferative Activity of Taxol Produced by Penicillium chrysogenum
Source: Molecules. 2020 Oct 20;25(20):4822. doi: 10.3390/molecules25204822 (PMC7588014; doi:10.3390/molecules25204822)
Supplement: Supplementary file 1 [file molecules-25-04822-s001.pdf]

Supplementary Table 1. Effect of different media on growth and taxol production by *P.chrysogenum*

Broth medium	Taxol µg/L	Fungal biomass g/L
**PD**	170a ± 3.0	20a ± 2.0
**ME**	85.63d ± 10.0	10b ± 3.0
**YSE**	110c ± 10.0	15a ± 5.0
**CD**	130.72b ± 10.0	17a ± 2.0

Potato dextrose (PD) (Zhang et al. 2009) (g L^{-1}): potato infusion 200, D-glucose 20 . **Malt extract agar medium (ME) (Bilgrami and Verma, 1981) composed of (g/L):** 20.0, malt extract; 5.0 ,yeast extract ;20.0, agar agar and 1000 ml, distilled H_2O**. yeast Sucrose-extract (YSE) (Scott et al., 1970) Composed of (g/L)**:40.0,sucrose ; 20.0 ,yeast extract ;15.0, agar-agar and 1000ml, distilled H_2O. **C`zapek`s Dox medium (CD) (Oxoid, 1982) composed of (g/L):** 30.0, sucrose; 3.0, $NaNO_3$; 1.0 ,KH_2PO_4; 0.5, $MgSO_4.7H_2O$;0.5, KCl; 0.01, $FeSO_4.7H_2O$ and 20.0, agar-agar and 1000 ml distilled H_2O. Calculated mean is for triplicate measurements from two independent experiments ± SD; means with different letters in the same column are considered statistically different (LSD test, $p < 0.05$)
